# Ambient Oxygen Concentration Enrichment When Using a Face Mask Connected to the Breathing Circuit Versus a Venturi Device: A Simulation Study

**DOI:** 10.7759/cureus.91195

**Published:** 2025-08-28

**Authors:** Danielle B Horn, Ken D Nguyen, Joni M Maga, Richard H Epstein

**Affiliations:** 1 Pain Medicine, Jackson Memorial Hospital, Miami, USA; 2 Medical Education, University of Miami Miller School of Medicine, Miami, USA; 3 Anesthesiology, University of Miami Miller School of Medicine, Miami, USA

**Keywords:** equipment safety, fire risk, mask, oxygen inhalational therapy, simulation

## Abstract

Background

The anesthesiology standard of care requires limiting oxygen (O_2_) concentration to less than or equal to 30% during monitored anesthetic care for upper chest and head and neck surgery to reduce the fire risk. While Venturi devices are used to regulate O_2_ concentration, they generate high flow rates, which may increase O_2_ concentration near the face mask. Even small increases of O_2_ concentration greater than room air significantly increase combustibility, highlighting the need for careful O_2_ delivery strategies.

Methods

A face mask was attached to a full-body patient simulator. The mask was connected to 1) the breathing circuit via a 15-mm tracheal tube adapter using the anesthesia machine’s O_2_ blender set at 30%, or 2) the auxiliary O_2_ port using a 31% Venturi device. Surgical drapes were placed on intravenous poles (“ether screen”) to separate the anesthesia and surgical side. Inflow O_2 _flow was 6 L/min for both groups, with the Venturi delivering ≈52 L/min. After room air calibration, ambient O_2_ concentrations were measured at three sites on the anesthesia side and three on the surgical side of the drape at no flow and then 15, 30, and 60 minutes after O_2_ flow initiation. Experiments were repeated three times.

Results

The pooled differences in the O_2_ concentration increases between the two devices were 0.80% (95% CI 0.53 to 1.07, P<.001) on the surgical side and 2.22% (95% CI 2.02 to 2.42, P<.001) on the anesthesia side of the ether screen. At 60 minutes, the mean (SD) concentrations in the Venturi group were 22.99% (0.31%) on the anesthesia side and 21.72% (0.90%) on the surgical side. Corresponding values in the anesthesia circuit group were 21.07% (0.12%) and 21.00% (0.11%).

Conclusions

A 31% Venturi device resulted in clinically relevant increases in ambient O_2_ concentrations on both sides of the ether screen compared to using the anesthesia machine O_2_ blender set at 30%. The differences are substantive because each 1% increase in the ambient O_2_ concentration increases the combustion rate of cotton by 15%. The use of a Venturi device to regulate O_2_ concentrations in the presence of an ignition source during head and neck surgery is not recommended. We advocate for anesthesia machine manufacturers to provide an integrated O_2_ blender for the auxiliary gas outlet of all their products as a fire safety measure.

## Introduction

During monitored anesthesia care for upper chest and head and neck surgery in the presence of an ignition source (e.g., an electrosurgical device), current guidelines recommend that the delivered oxygen (O_2_) concentration should be limited to no greater than or equal to 30% to mitigate the fire risk [1-4]. We recently described a method for delivering O_2_ using the anesthesia machine's O_2_ blender and breathing circuit. In this approach, a nasal cannula or face mask is connected to the Y-piece of the circuit via a 15-mm adapter from a 5.0 mm tracheal tube. The adjustable pressure-limiting (APL) valve is partially closed to generate circuit pressure, which influences the delivered flow rates [5]. Loeb previously suggested using a Venturi device to regulate the O_2_ concentration [6], but such devices generate high flow rates, raising concern about increasing the O_2_ concentration near the mask more than when using a face mask at conventional flow rates. For example, based on the device manufacturers' standard entrainment ratios, a 31% Venturi device requiring 6 L/min O_2_ flow will entrain 45.5 L/min of room air, producing a total flow of 51.5 L/min. It is a misconception that an ambient O_2_ concentration of 30% is without increased risk compared to room air. Even small increases above 21% produce an increase in combustibility, with the burn rate of cotton rising linearly from 2 cm/sec in a 21% O_2_ environment to approximately 5 cm/sec when the concentration is increased to 31% [7]. Because cotton is a common component of surgical drapes and dressings in the operative field, a higher ambient O_2_ concentration may significantly increase the speed at which these materials ignite and burn, elevating the risk of surgical fires. Because Venturi devices generate high flow rates, they may saturate the nearby environment with O_2_-enriched gas, particularly in the presence of surgical drapes that limit dispersion. In contrast, face masks connected to the anesthesia circuit using the O_2_ blender operate at lower total flow rates. For this reason, we hypothesized that differences in the ambient O_2_ concentration near the face mask would be >0.5% higher when using a Venturi device than a face mask connected to the circuit at comparable delivered O_2_ concentrations. We selected 0.5% as the threshold based on its potential clinical significance in increasing fire risk [3,7].

## Materials and methods

Institutional review board approval was not indicated because this was a laboratory investigation not involving patients, animals, or subjects. This manuscript adheres to the Strengthening the Reporting of Observational Studies in Epidemiology (STROBE) guidelines.

Experimental design

**Figure 1:**
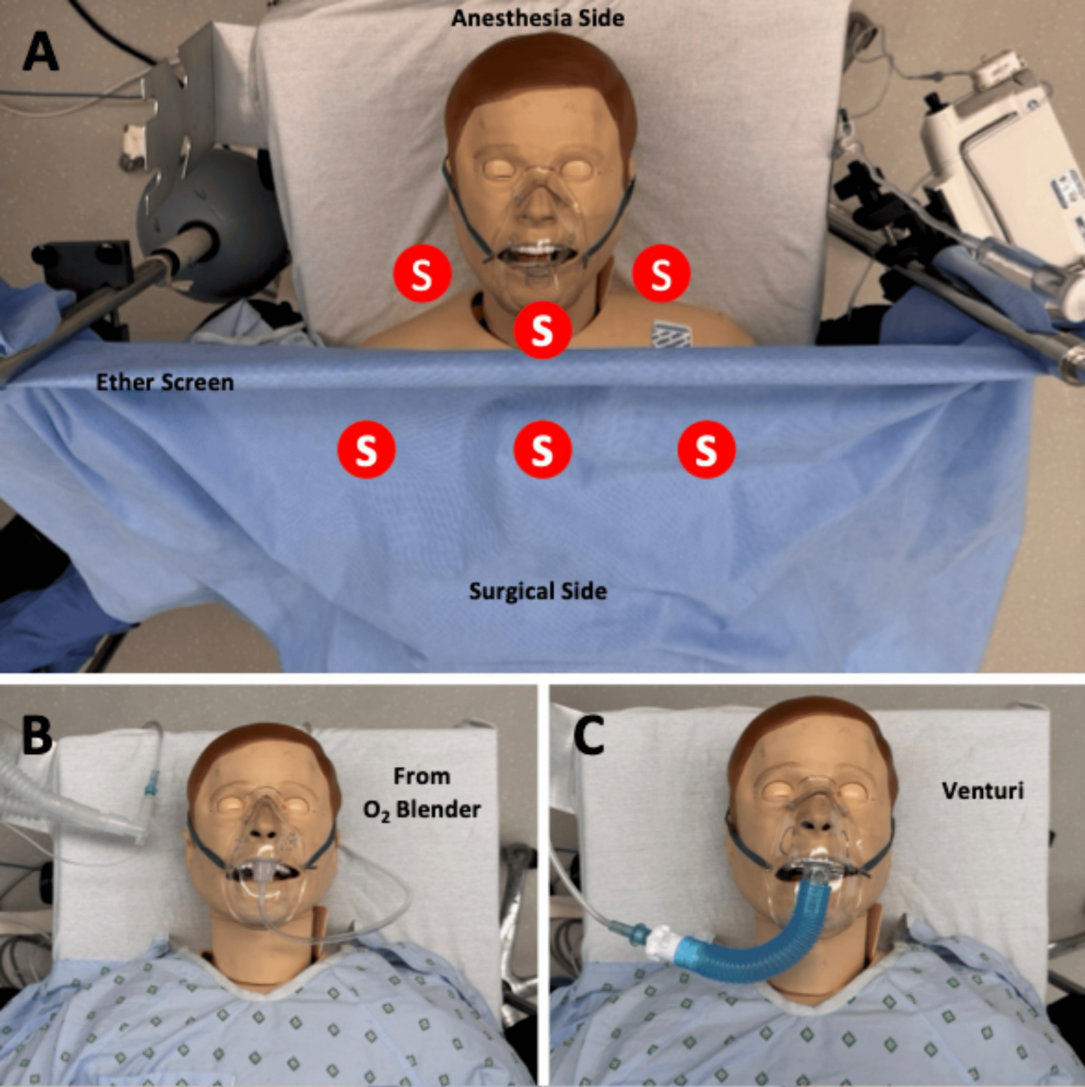
Photograph of the experimental setup In the upper panel (A), the sampling sites are noted by the red circles, labeled with an S. An ether screen, lowered in the photo from the 5-foot height used during the experiments, separated the anesthesia and the surgical side. Those on the anesthesia side were approximately 6 inches equidistant from the center of the mask, whereas those on the surgical side were just beyond the ether screen at the center and in line with the shoulders. The bottom left panel (B) shows the connection of the supply tubing to the face mask to the anesthesia circuit using a 15-mm adapter from a 5.0 mm tracheal tube inserted into the tubing. The oxygen (O_2_) concentration was regulated using the anesthesia machine O_2_ blender. The bottom right panel (C) shows the connection of the Venturi device. The ether screen was removed in panels B and C for visualization.

The simulation experiments were conducted on September 17, 2024, at the Center for Patient Safety (CPS), a joint venture of the University of Miami Miller School of Medicine and the Jackson Health System. In the CPS operating room, a SimMan® Essential Bleeding patient simulator (Laerdal, Wappingers Falls, NY, USA) was used on an operating room table with the face mask secured by a strap. A standard, disposable surgical drape was fastened between two poles at a height of approximately 5 feet on either side of the patient simulator (i.e., an “ether screen”), separating the testing area into two zones. We refer to the area between the anesthesia machine and the drape as the anesthesia side of the ether screen, corresponding to the area surrounding the face mask. We refer to the area on the other side of the drape as the surgical side of the ether screen. The face mask was connected to the anesthesia circuit using the 15-mm adapter from a 5.0 mm tracheal tube, as previously described [5], or to a Venturi device (Vyaire Medical, Inc., Mettawa, IL, USA). A Datex-Ohmeda Aisys anesthesia machine (GE HealthCare Technologies, Chicago, IL, USA) was used with its digital O_2_ blender set to 30% and a 6 L/min flow rate for experiments using the anesthesia circuit as the delivery portal. A 31% Venturi device was used with an O_2_ flow rate of 6 L/min for the Venturi experiments. An annotated photograph of the experimental setup is shown in Figure 1.

O_2_ concentrations were measured with an Expedition-X Oxygen Analyzer (OxyCheq, Marianna, FL, USA), calibrated in room air to 20.9% before each set of replicates. The analyzer concentrations were linear between 21% and 50% (slope =1.00, intercept = 0.00, Pearson R=0.999), and the 98% response time in 50% O_2_ (i.e., to 48%) was 9.05 sec (standard deviation 0.40 sec). For each ambient O_2_ measurement, the sensor was allowed to stabilize for 60 seconds before recording the value. Baseline measurements were made near the face mask without O_2_ flow at the start of each sequence of recordings. Additionally, prior to each sequence of measurements, the baseline O_2_ concentration inside the mask was measured for each O_2_ level (30%, 40%, and 50%) to confirm the accurate delivery of the intended O_2_ concentration. Simulations with the anesthesia circuit or Venturi device were conducted three separate times, with each experiment lasting one hour. During each sequence, measurements were made at 15, 30, and 60 minutes after the start of O_2_ flow at the center, right, and left of the midline adjacent to the surgical side of the drape and on the anesthesia side of the drape at a radial distance of approximately 6 inches from the center of the face mask at angles of 0°, 90°, and 180°.

Differences between 20.9% and the measured values were computed to determine the increases in the ambient O_2_ concentration from baseline.

Statistical analysis

Analysis of variance was performed using Stata v18.5 (College Station, TX, USA), followed by Tukey’s honestly significant difference test for comparative measurements if the ANOVA F value was statistically significant at P<0.05.

Based on a concentration of 20.9% O_2_ in room air and a standard deviation of 0.1%, as measured during 15 room air determinations, a sample size of 6 would be required to demonstrate at least a 0.5% increase in the concentration at P <0.05 using a two-sided, two-sample t-test comparing independent means.

## Results

Averages of the nine replicates at each combination of measurement location, delivery device, and time are presented in Table 1.

**Table 1:** Oxygen concentrations by delivery device, location, and time ^a^ There were N=9 measurements at each combination of side, device, and time.

		% Ambient oxygen, mean (standard deviation)^a^			
Ether Screen Side	Delivery Device	Baseline	15 min	30 min	60 min
Anesthesia	Anesthesia Circuit	20.92 (0.07)	20.94 (0.07)	21.11 (0.12)	21.07 (0.12)
	Venturi	20.84 (0.07)	23.20 (0.53)	23.59 (0.47)	22.99 (0.31)
Surgical	Anesthesia Circuit	20.88 (0.04)	20.91 (0.03)	20.94 (0.05)	21.00 (0.11)
	Venturi	20.88 (0.11)	21.84 (0.58)	21.68 (0.58)	21.72 (0.90)

At the anesthesia side of the ether screen, in the anesthesia circuit group, the ambient O_2_ concentrations adjacent to the face mask increased from 20.92% at time 0 (baseline) to 21.07% at 60 minutes. In the Venturi device group, concentrations increased from 20.84% to 22.99%.

At the surgical side of the ether screen, in the anesthesia circuit group, the ambient O_2_ concentrations increased from 20.88% at baseline to 21.00% at 60 minutes. In the Venturi device group, concentrations increased from 20.88% to 21.72%.

**Figure 2:**
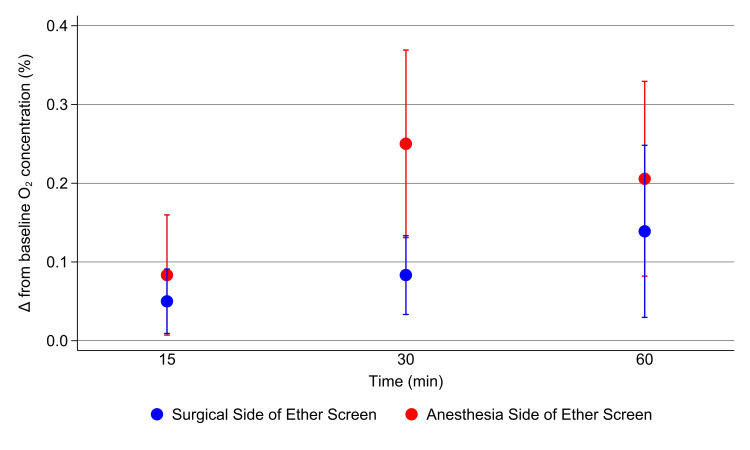
Change from baseline oxygen concentration using a face mask connected to the anesthesia circuit Mean differences between the baseline (room air) O_2_ concentration and measurements at the surgical and anesthesia side of the ether screen near the drape and the face mask during administration of 30% O_2_ by face mask at a 6 L/min flow rate via the anesthesia circuit. The error bars represent the standard deviations. Comparison p-values derived from data in Figures 2, 3 are described in the text below.

**Figure 3:**
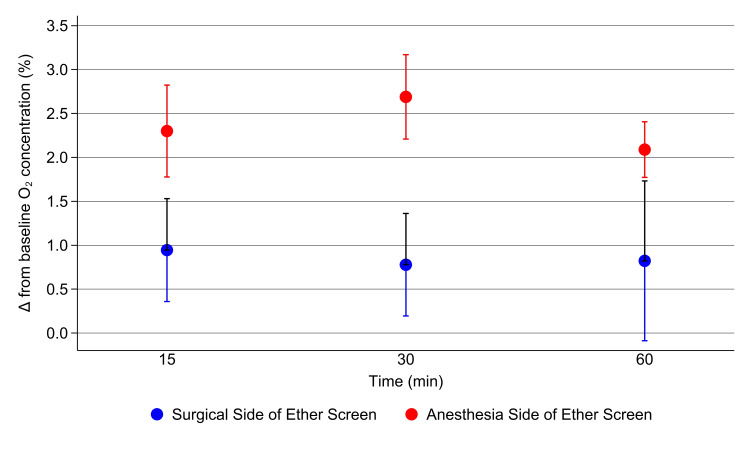
Change from baseline oxygen concentration using a Venturi device Differences between the baseline (room air) O_2_ concentration and measurements at the surgical and anesthesia side of the ether screen near the drape and the face mask, respectively, during the administration of O_2_ by face mask via a 31% Venturi device connected to the auxiliary gas outlet on the anesthesia machine set at 6 L/min of 100% O_2_. The error bars represent the standard deviations. Comparison p-values derived from data in Figures 2, 3 are described in the text below.

By two-way analysis of variance, there was no interaction between the time of measurement after the start of gas flow within each delivery device group and side of the ether screen and the increase in the O_2 _concentration from baseline at 15, 30, and 60 minutes (Figures 2, 3).

Differences between the two measuring sites (the surgical versus the anesthesia side of the ether screen) were significant (P<.001). Therefore, the three replicated time values at the three measurement times (n=9 values) were pooled, and the differences in the increases from baseline for each delivery device were compared for each side of the ether screen. At the surgical side of the screen, the mean difference in the O_2_ concentration increase was 0.80% (95% CI 0.53% to 1.07%, P<.001, Figure 2). At the anesthesia side of the ether screen (i.e., adjacent to the face mask), the mean increase was 2.22% (95% CI 2.07% to 2.42%, P<.001).

## Discussion

Using the Venturi device resulted in a substantively greater (mean 2.2%) increase in the O_2_ concentration on the anesthesia side of the ether screen adjacent to the face mask over the baseline value compared to using the anesthesia circuit when delivering comparable concentrations of O_2_. This enhancement of the ambient O_2_ concentration has important ramifications when the surgical site is close to the face mask and an ignition source is used. The increase on the surgical side of the ether screen was less dramatic (mean 0.8%) but substantive because even small increases in ambient O_2_ can significantly increase fire risk. For example, cotton, a common material in surgical drapes and dressings, becomes more flammable in oxygen-enriched environments. Each 1% increase in the ambient O_2_ concentration increases the rate of combustion of cotton by 15% as compared to room air (up to ambient O_2_ concentrations of 35%) [7]. During the experiments, we were careful to maintain the integrity of the ether screen, but in clinical practice, that might not always be the case, with the potential for more O_2_ leakage into the surgical field. Thus, the 0.8% increase observed on the surgical side of the ether screen should be considered conservatively low. The nominal 1% difference between the delivered O_2_ concentrations cannot account for the large difference on the anesthesia side of the screen because even a 40% delivered concentration via the anesthesia circuit at 6 L/min only increased the local ambient concentration at the same distance from the center of the face mask used during the experiments by 0.60% (95% CI 0.53% to 0.67%, unpublished data). We emphasize that an ambient concentration of 30% O_2_ is not inherently safe; any increase above 21% substantially increases the combustion rate should a fire occur [7].

In our previous report, using the anesthesia circuit to deliver a regulated O_2_ concentration from the anesthesia machine O_2_ blender to a face mask is straightforward because high circuit pressure does not need to be achieved by closing the APL valve [5]. Because the consequences of an operating room fire are dire [1], preference for an alternative delivery method that increases the risk, even marginally, is questionable. We continue to advocate for anesthesia machine manufacturers to provide an O_2_ blender for the auxiliary gas outlet of all their products as a fire safety feature. Among the major producers, this feature became commercially available from Mindray (Mindray, North America, Mahwah, NJ) in 2011, followed by GE HealthCare (GE HealthCare, North America, Chicago, IL) in 2016 for some of their products. An integrated O_2 _blender is not currently available for Dräger anesthesia machines (Drägerwerk, Lübeck, Germany). A blender for the auxiliary gas port incorporated into the anesthesia machine is preferable to adapting O_2_ mask tubing to connect to the anesthesia circuit or connecting a Venturi device to a source delivering 100% O_2_. Venturi devices are not suitable when supplemental O_2 _needs to be administered via a nasal cannula because the high pressure from the tubing will limit the amount of entrained air and thus raise the O_2 _concentration generated by the device [8].

Limitations

Our study has several limitations. First, the tests were conducted in a simulated environment with a lower room air exchange rate than is typically provided in operating rooms [9]. This difference may have resulted in overestimating ambient concentrations measured in a clinical environment. Second, although our O_2_ analyzer had a fast response time (i.e., <10 seconds to 98% of the final value), it might have missed transient, localized increases in local O_2 _concentration. Consequently, our measurements should be considered as a minimum estimate of the actual concentrations that can occur when 30% O_2_ is delivered. Third, we did not test multiple Venturi devices from multiple manufacturers. Oxygen concentrations delivered will vary from the nominal concentration of the device, depending on the manufacturing tolerances of the orifice, and are also affected if an incorrect O_2_ flow rate is provided or if there is back pressure in the circuit distal to the device [6]. If the outflow from the Venturi device becomes inadvertently obstructed, for example, if it is located under the drapes, then room air will not be entrained and the 100% O_2_ feeding the Venturi device from the auxiliary O_2_ supply will spill out through the vents in the Venturi device, markedly increasing the local O_2_ concentration. These considerations create additional concern about using Venturi devices when there are fire safety risks compared to the expected, more uniform performance of digital O_2_ blenders on anesthesia machines.

## Conclusions

When supplemental O_2_ via a face mask is required during upper chest and head and neck surgical procedures with an ignition source, using a Venturi device to control the delivered O_2_ concentration generates additional, substantive fire risk compared to using the O_2_ blender from the anesthesia machine. While, ideally, that would be provided via an integrated blender controlling the O_2_ concentration supplied by the auxiliary gas outlet of the machine, the use of the anesthesia circuit and the standard machine blender provides a viable alternative to the regulation of the administered O_2_ concentration. Notwithstanding these considerations, when providing sedation for procedures at an increased risk of fire, avoiding supplemental O_2_ if not necessary, limiting the concentration to provide a minimally acceptable pulse oximetry saturation, and considering general anesthesia with a sealed airway should be considered part of the overall fire safety strategy.
